# Economic Evaluation of Contact Force Catheter Ablation for Persistent Atrial Fibrillation in the United States

**DOI:** 10.1016/j.hroo.2022.09.011

**Published:** 2022-09-20

**Authors:** Jose Osorio, Moussa Mansour, Daniel Melby, Ryan J. Imhoff, Tina D. Hunter, Sonia Maccioni, Tom Wei, Andrea Natale

**Affiliations:** ∗ Arrhythmia Institute at Grandview, Birmingham, Alabama; † Massachusetts General Hospital, Boston, Massachusetts; ‡ Minneapolis Heart Institute at Abbott-Northwestern Hospital, Minneapolis, Minnesota; § Real-World Evidence, CTI Clinical Trial and Consulting Services, Covington, Kentucky; || Franchise Health Economics and Market Access, Johnson & Johnson, Irvine, California; ¶ Texas Cardiac Arrhythmia Institute at St. David’s Medical Center, Austin, Texas

**Keywords:** Economic evaluation, Atrial fibrillation, Persistent atrial fibrillation, Catheter ablation, Cost-effectiveness, Radiofrequency ablation, Break-even analysis

## Abstract

**Background:**

Atrial fibrillation (AF) is the most prevalent cardiac arrhythmia, and it increases the risk of stroke, heart failure, and other cardiac complications. Catheter ablation is well-established as a treatment for paroxysmal AF, and the recent PRECEPT (Prospective Review of the Safety and Effectiveness of the THERMOCOOL SMARTTOUCH SF Catheter Evaluated for Treating Symptomatic Persistent AF) clinical trial resulted in the catheter gaining approval for the treatment of persistent AF in the United States.

**Objectives:**

To construct an economic simulation model, based on the results of the PRECEPT trial, to monetize the impact of radiofrequency catheter ablation (RFCA) compared with medical therapy (MT).

**Methods:**

Cost-offset and break-even analyses were performed to assess the economic impact of RFCA vs MT for adult persistent AF patients. Three perspectives were considered: commercial payers, Medicare, and self-insured employers. A cohort-level decision tree model was developed and validated in TreeAge Pro 2019. Sensitivity analyses were performed to determine the robustness of findings.

**Results:**

For all 3 types of payer, RFCA had a higher initial cost compared with MT. However, reductions in health care utilization after ablation, driven by decreased cardiovascular hospitalizations, led to an annual cost offset of between $5037 and $8402 after the first year. Projecting this forward resulted in an estimated cost break-even after 5.9, 4.2, and 5.1 years for commercial payers, Medicare, and self-insured employers, respectively.

**Conclusion:**

In addition to providing clinical benefits, RFCA may be a valuable economic investment for U.S. payers, substantially reducing utilization after the first year.

**Key Findings**

▪ Despite a higher initial cost, radiofrequency catheter ablation led to reduced health resource utilization moving forward, corresponding to annual cost offset of between $5037 and $8402.
▪ Projecting the annual cost offset forward, the investment in a radiofrequency catheter ablation procedure is projected to break even after approximately 4 to 6 years for U.S. payers.
▪ The largest driver of the time to break even is the reduction in cardiovascular hospitalizations after radiofrequency catheter ablation, accounting for roughly half of the annual cost offset value for payers.

## Introduction

Atrial fibrillation (AF) is estimated to affect between 2.7 and 6.1 million people in the United States.^1^ AF can lead to a significant increase in health care utilization, particularly due to an increased risk of stroke, heart failure, and other cardiac complications.^2^ In addition to the physical burden for patients, there are substantial costs associated with the treatment of AF. One report estimated annual U.S. inpatient, emergency room, and outpatient costs to be $6.65 billion in 2005 U.S. dollars (USD), equivalent to approximately $9.11 billion in 2021 USD.^3^ While episodes of AF may be short-lived and infrequent for patients with early paroxysmal AF, a significant proportion of patients progress to more sustained arrhythmias, such as persistent AF (PsAF).^4^ Strong evidence suggests that patients with PsAF are at a higher risk of stroke, heart failure, and death, compared with those with paroxysmal AF.^2^

Catheter ablation (CA) for the treatment of AF has been shown to be associated with greater reductions in AF recurrence, AF-related complications, and health resource utilization when compared with medical therapy (MT) and, specifically, antiarrhythmic drugs (AADs).^5^, ^6^, ^7^, ^8^ CA for the treatment of PsAF is commonly used in real world practice. In fact, one European survey involving 72 sites across 10 countries reported nearly one-third of CA procedures performed were for PsAF patients.^9^

The recent PRECEPT (Prospective Review of the Safety and Effectiveness of the THERMOCOOL SMARTTOUCH SF Catheter Evaluated for Treating Symptomatic Persistent AF) trial (https://clinicaltrials.gov/ct2/show/NCT02817776) resulted in the catheter indication by the U.S. Food and Drug Administration specifically for the treatment of PsAF patients across the disease spectrum (THERMOCOOL SMARTTOUCH SF Catheter; Biosense Webster, Irvine, CA). The PRECEPT trial was a prospective, multicenter, single-arm, nonrandomized, Investigational Device Exemption clinical study that demonstrated the safety and effectiveness of contact force–guided radiofrequency CA (RFCA) for the treatment of PsAF. Findings included an 80.4% rate of freedom from any documented symptomatic atrial arrhythmia at 15 months postprocedure.^10^ Additionally, 84.2% of patients experienced freedom from cardiovascular hospitalization at 15 months postprocedure, and 69.2% of patients were no longer on AADs at 15 months postprocedure, indicating a significant reduction in health care utilization postprocedure.^11^

The objective of the current study was to construct an economic simulation model, based on the results of the PRECEPT trial, to quantify the economic impact of RFCA compared with MT for the treatment of PsAF.

## Methods

### Overview

Cost-offset and break-even analyses were performed to assess the economic impact of RFCA vs MT for adult PsAF patients that had previously failed at least 1 AAD. The MT included patients on any class I to IV AAD. Analyses were performed from the perspective of 3 different U.S. health care payers: (1) commercial payers, (2) Medicare, and (3) self-insured employers. This model utilized de-identified patient data from the PRECEPT clinical trial as the primary data source to construct and inform analysis. The methods and patient selection criteria of the PRECEPT clinical trial, along with the primary efficacy and safety results, have been previously published,^10^ as have the impacts on quality of life and symptom burden.^11^ Supplementary data sources included published literature,^5^^,^^12^, ^13^, ^14^, ^15^, ^16^ publicly available data, and government reports^17^, ^18^, ^19^, ^20^, ^21^, ^22^, ^23^, ^24^, ^25^ for the estimation of model parameters that were not collected during the PRECEPT trial.

### Model structure

A cohort-level decision tree model was developed to simulate the experience of PsAF patients after being treated with RFCA or MT. The model selection was based on the nature of the outcomes and the short time horizon. The structure and design of the model was based on published guidelines,^26^ as well as on the input from experienced clinicians in the fields of cardiology and electrophysiology. The decision tree was developed and validated in TreeAge Pro 2019 (TreeAge Software, Williamstown, MA), with costs expressed in 2020 U.S. dollars (USD). This analysis is based primarily on previously published data, though supplementary analyses were performed using additional de-identified data from patients in the PRECEPT trial that already consented to the use of their trial data; thus, this study is exempt from institutional review board review.

**Figure 1:**
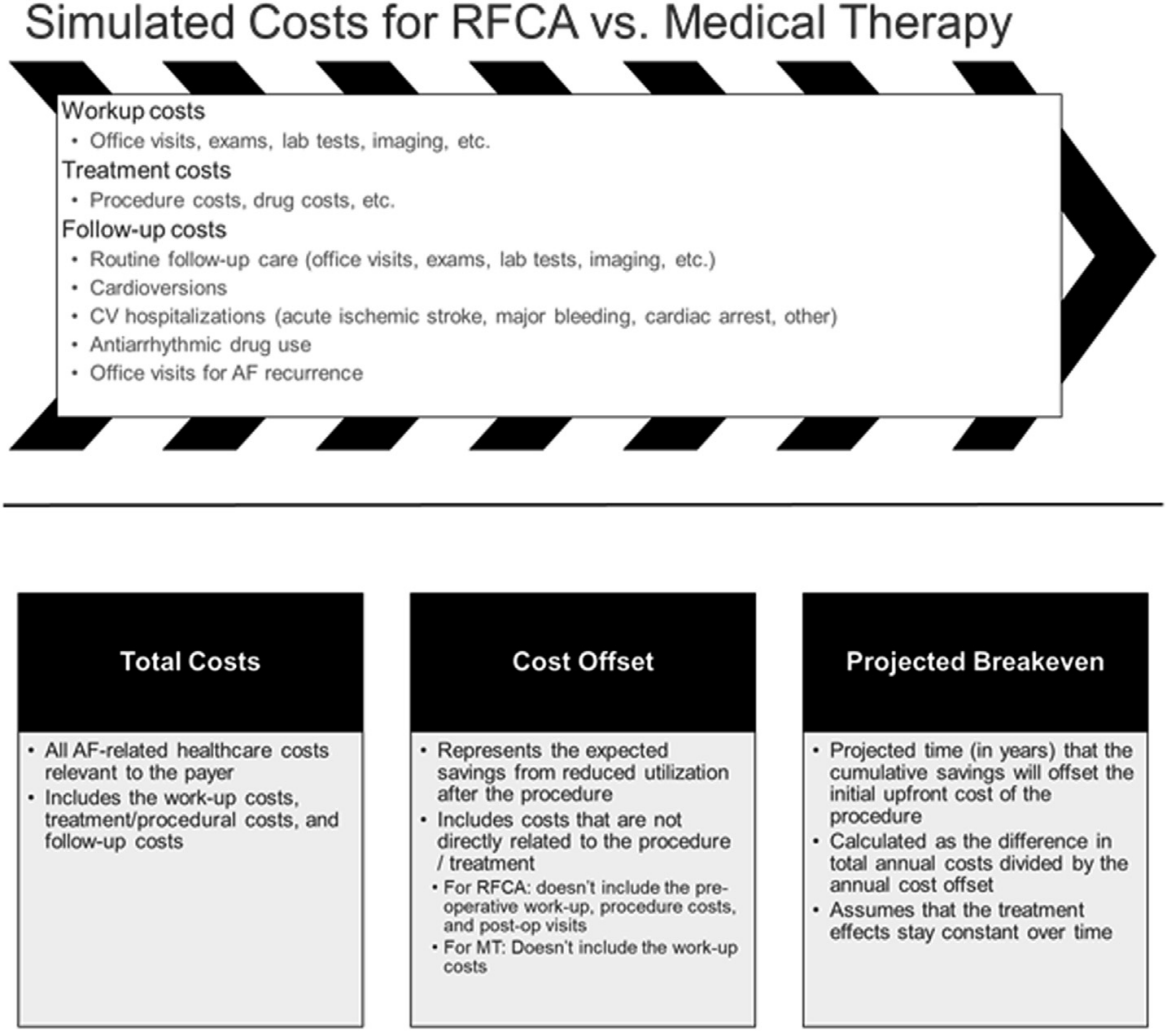
Study overview. Patient experience was simulated for 1 year after initiating treatment, to estimate the cost of the pretreatment workup, treatment cost, and related follow-up costs. Utilization measures during follow-up comprised routine follow-up visits, cardioversions, cardiovascular (CV) hospitalizations, antiarrhythmic drug (AAD) usage, and atrial fibrillation (AF) recurrence. The model assessed 3 primary outcomes: total annual health care costs, annual cost offset, and the projected break-even point. MT = medical therapy; RFCA = radiofrequency catheter ablation.

An overview of the current study, including costs considered and model outcomes, is shown in Figure 1. The patient journey was simulated for the first year after initiating treatment and included the cost of the pretreatment workup, treatment cost, and related follow-up costs. Utilization measures throughout follow-up comprised routine follow-up visits, cardioversions, cardiovascular (CV) hospitalizations, AAD usage, and AF recurrence. To simplify the analysis and keep comparisons straightforward, the model did not allow treatment crossover from MT to ablation, and repeat ablations were not considered. A subset of patients was assumed to remain on AADs postablation, according to the results from the PRECEPT trial.

The model assessed 3 primary outcomes: total annual health care costs, annual cost offset, and the projected break-even point. Total annual costs included all AF-related health care costs relevant to the payer. The annual cost offset was calculated as the expected savings from reduced utilization after the procedure, including costs not directly related to the initial procedure (excluded workup costs, procedure costs, and postprocedure follow-up that would not continue beyond the first year). The break-even point was defined as the projected time (in years) that the cumulative savings offset the initial upfront cost of the ablation procedure. This was calculated as the difference in total annual costs divided by the annual cost offset. It was assumed that treatment effects were constant over time.

### Clinical and utilization data

Clinical and utilization data for the RFCA arm were populated using the PRECEPT trial data. As the trial was single arm in nature, it was assumed that data collected at baseline or preablation were also reflective of patients on MT who would have been eligible for CA (ie, the comparator group). Preablation CV hospitalization and AF episodes were not collected during the PRECEPT trial, and consequently, a best-evidence literature review was conducted to provide supplementary data. A systematic literature review and meta-analysis of RFCA vs MT for PsAF patients was selected for estimates of the rates in the MT arm.^5^ The relative risk ratios published in this meta-analysis were applied to the RFCA cohort estimates from the PRECEPT trial to estimate these outcomes for the MT arm.

A summary of clinical and utilization inputs is presented in Table 1. Clinical outcomes were assumed to be identical for commercial payers and self-insured employers. However, the estimates for the Medicare population were obtained from a subanalysis of the PRECEPT trial data based on patients 65 years of age or older. Clinical outcomes included cardioversions, AAD usage, AF recurrence, and CV hospitalizations. CV hospitalizations were subdivided into 4 categories: acute ischemic stroke, major bleeding events, cardiac arrest, and all other or general CV hospitalizations. To estimate the differences in specific event rates, a large retrospective claims database analysis that assessed the generalizability of the Catheter Ablation vs Antiarrhythmic Drug Therapy for Atrial Fibrillation (CABANA) clinical trial was used.^14^ Relative risk ratios from this analysis were multiplied by the rates observed in the PRECEPT trial RFCA cohort to estimate the MT arm.

**Table 1:** Clinical and utilization inputs

Parameter	Commercial/self-insured employers		Medicare	
	CA	MT	CA	MT
AAD usage, %				
Months 1–3				
Class I/III	42.8	97.0	41.2	97.8
Class II/IV	5.2	12.3	5.2	10.8
Months 4–6				
Class I/III	42.8	97.0	41.2	97.8
Class II/IV	5.2	12.3	5.2	10.8
Months 7–9				
Class I/III	30.5	97.0	30.5	97.8
Class II/IV	5.1	12.3	4.7	10.8
Months 10–12				
Class I/III	42.8	97.0	41.2	97.8
Class II/IV	5.2	12.3	5.2	10.8
Mean number of cardioversions per year	0.231	1.917	0.280	1.994
Probability of recurrence in first year	0.265	0.552	0.287	0.597
Mean number of CV-related hospitalizations per year	0.156	0.289	0.184	0.341
Stroke	0.004	0.008	0.005	0.009
Bleeding event	0.013	0.012	0.015	0.015
Cardiac event	0.000	0.002	0.000	0.002
Other	0.139	0.267	0.164	0.316
Missed number of days from work (per event)				
Office visit	0.5	0.5	N/A	N/A
Cardioversion	1.5	1.5	N/A	N/A
Ablation/reablation	5.0	5.0	N/A	N/A
CV events				
Stroke	146.8	146.8	N/A	N/A
Bleeding event	91.3	91.3	N/A	N/A
Cardiac arrest	77.8	77.8	N/A	N/A
Other	3.5	3.5	N/A	N/A

AAD = antiarrhythmic drug; CA = catheter ablation; CV = cardiovascular; MT = medical therapy; N/A = not applicable.

Finally, the number of missed days from work was estimated for the perspective of the self-insured employer. Each instance of utilization within the model was assigned the average number of days that a patient would be expected to miss work. For acute ischemic stroke, major bleeding events, and cardiac arrest, it was assumed that the event occurred in the middle of the model year (ie, at the 6-month time point) in the simulation. Consequently, a patient could miss up to a maximum of 6 months of work. Estimates for each event are shown in Table 1.

### Costs

Costs incurred for each treatment were estimated over a 1-year time horizon, and were based on figures reported by the U.S. Bureau of Labor Statistics,^24^^,^^25^ using the Medical Care component of the U.S. Consumer Price Index to adjust for inflation.^25^ Only disease-related costs that would be incurred by payers and that were different across cohorts were included in this analysis. According to a meta-analysis by Chen and colleagues,^5^ the difference in complication or adverse event rates between RFCA and MT was not statistically significant; therefore, it was not included in the model. Costs specific to each payer were used, with commercial and self-insured employer medical costs assumed to be identical. Published conversion factors were used to determine appropriate costs for each payer type.^27^ For the commercial and Medicare payer models, only direct medical costs were included. For the self-insured employer model, costs associated with missed time from work were estimated by multiplying the expected number of days missed from work by the average salary for an adult worker in the United States, according to the U.S. Bureau of Labor Statistics.^24^

A summary of cost inputs is presented in Table 2. These costs included pretreatment costs, treatment costs, and follow-up costs. Pretreatment costs comprised a diagnostic workup, testing, or consultation fees. Treatment costs were related to the procedure for the RFCA cohort and related to medication for the MT cohort. Follow-up costs included the costs of routine monitoring, cardioversions, AAD usage, AF recurrence, and CV hospitalizations. For acute ischemic stroke, major bleeding events, and cardiac arrest, the costs included all expected care in the first year, such as the index visit, follow-up, rehabilitation, and long-term-care facilities. These costs were estimated using published analyses of comprehensive care costs.^12^^,^^15^

**Table 2:** Cost inputs

Costs	Commercial/self-insured employers		Medicare	
	CA	MT	CA	MT
Pretreatment	$441	$220	$295	$173
Procedure	$40,975	N/A	$20,699	N/A
Follow-up				
Monitoring	$839	$801	$574	$635
AAD (per 3 mo)				
Class I/III	$251	$251	$193	$193
Class II/IV	$175	$175	$89	$89
Cardioversion (each)	$1119	$1119	$722	$722
AF recurrence episode (each)	$276	$276	$201	$201
Repeat ablation (each)	$40,975	N/A	$20,699	N/A
CV-related events (each)				
Stroke	$92,772	$92,772	$55,552	$55,552
Bleeding event	$58,205	$58,205	$34,853	$34,853
Cardiac event	$72,942	$72,942	$43,678	$43,678
Other	$25,452	$25,452	$15,241	$15,241
Missed days from work (self-insured employers only)	$206	$206	N/A	N/A

AAD = antiarrhythmic drug; CA = catheter ablation; CV = cardiovascular; MT = medical therapy; N/A = not applicable.

### Sensitivity analyses

A 1-way deterministic sensitivity analysis was conducted to identify which model parameters were most impactful to the results.^28^ Parameters were varied in a stepwise manner over a range of ±25% of the base value to observe the subsequent effects on results. A Monte Carlo probabilistic sensitivity analysis was conducted to assess the robustness of the model results and gauge the impact of uncertainty within the model.^28^ Each model parameter was represented by a distribution around the point estimate. Simulated scenarios randomly sampled each parameter distribution to obtain a full set of parameters, after which model outputs were calculated and stored for each iteration. The model was simulated for 100,000 iterations, each with a different set of parameter input values.

### Scenario analyses: Inclusion of repeat ablation and MT-to-ablation crossover

A scenario analysis was conducted to evaluate the assumption within the model that excludes repeat ablations, as well as the exclusion of patients in the MT arm receiving an ablation. To evaluate the model with these 2 aspects incorporated into the analysis, a repeat ablation rate, as well as an MT-to-ablation crossover rate, was introduced into the model. Ablation patients were already able to receive AADs after ablation in the base model; thus, crossover from that direction was already accounted for.

The repeat ablation rate was estimated using the PRECEPT trial data; however, as the PRECEPT trial was single arm and thus did not include MT crossover, the rate presented in the CABANA clinical trial was used, as it represented the highest-quality evidence available.^29^ The rate reported from the CABANA trial was over the full duration of follow-up, which had a mean of approximately 4 years. In order to interpolate this to an annual rate, the exponential decay formula was applied to determine the 1-year rate.

## Results

### Model results

**Figure 2:**
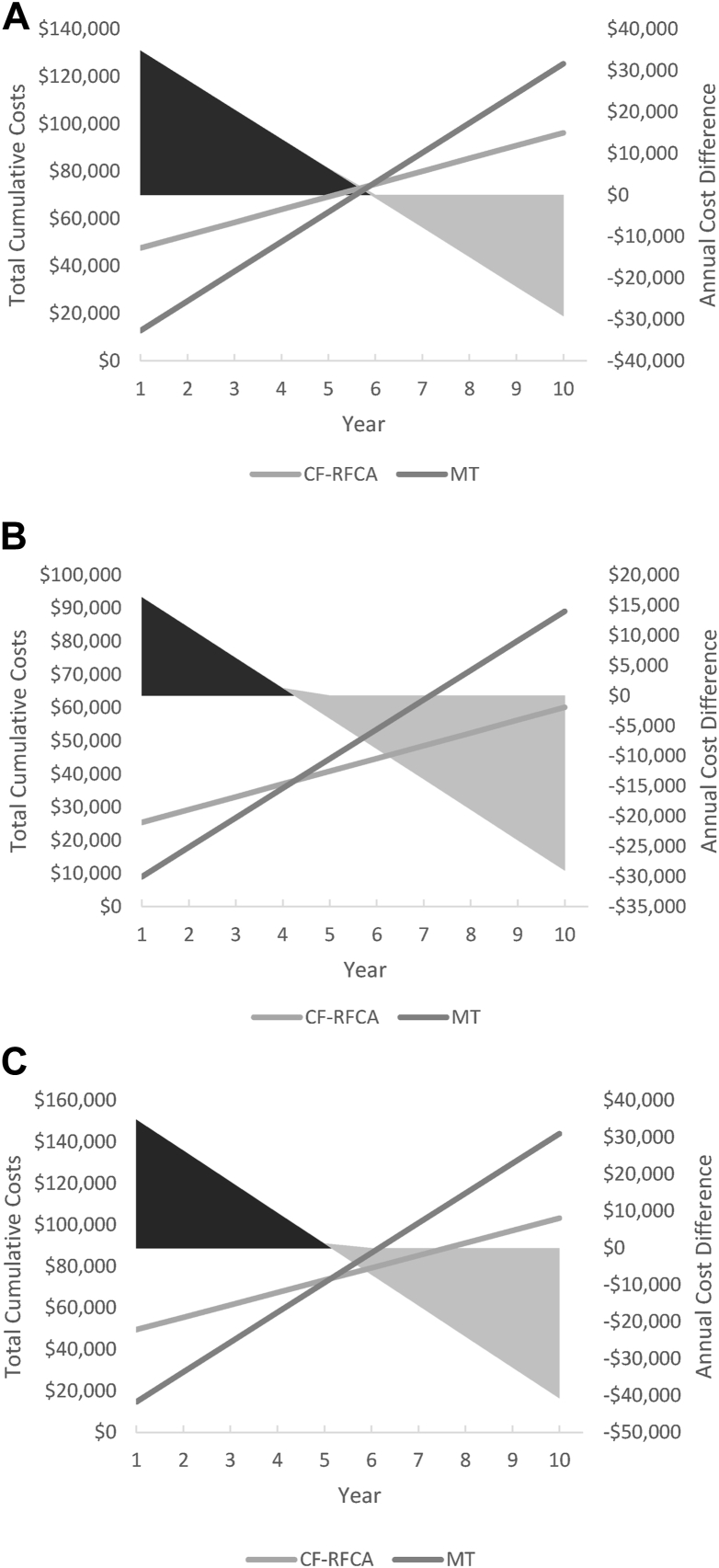
Summary of model results and break-even analysis. Despite an increase in up-front costs, radiofrequency catheter ablation (RFCA) led to reduced utilization over time, resulting in an annual cost offset or savings that was valued at between $5037 and $8402, depending on the payer group. Extrapolating this annual cost offset to future years, RFCA was expected to break even after 4.2, 5.1, and 5.9 years for A: commercial payers, B: Medicare, and C: self-insured employers, respectively. CF = contact-force; MT = medical therapy.

From the perspective of a commercial payer, the total expected annual costs are $47,652 for RFCA and $12,736 for MT (Table 3). The annual cost offset, representing the reduced utilization after RFCA, is valued at an annual savings of $7118 per year. Projecting this forward, the expected break-even point for cost is 5.9 years after the index treatment (Figure 2A). From the perspective of Medicare, the total annual costs are $25,421 for RFCA and $9063 for MT (Table 3). The annual cost offset is a savings of $5037 per year, with a projected break-even point of 4.2 years after RFCA (Figure 2B). From the perspective of a self-insured employer, the total annual costs were $49,554 for RFCA and $14,687 for MT (Table 3). The reduced utilization after RFCA is expected to lead to a cost offset of $8402 per year, with a projected break-even point for RFCA of 5.1 years for a self-insured employer (Figure 2C).

**Table 3:** Summary of model results

Strategy	Total cost	Incremental value	Offset/utilization only	Incremental value	Years until break-even
Commercial					
CA	$47,652	$34,916	$5398	–$7118	5.9
MT	$12,736	—	$12,516	—	—
Medicare					
CA	$25,421	$16,358	$3852	–$5037	4.2
MT	$9063	—	$8890	—	—
Self-insured employer					
CA	$49,554	$34,867	$5963	–$8402	5.1
MT	$14,687	—	$14,364	—	—

CA = catheter ablation; MT = medical therapy.

**Figure 3:**
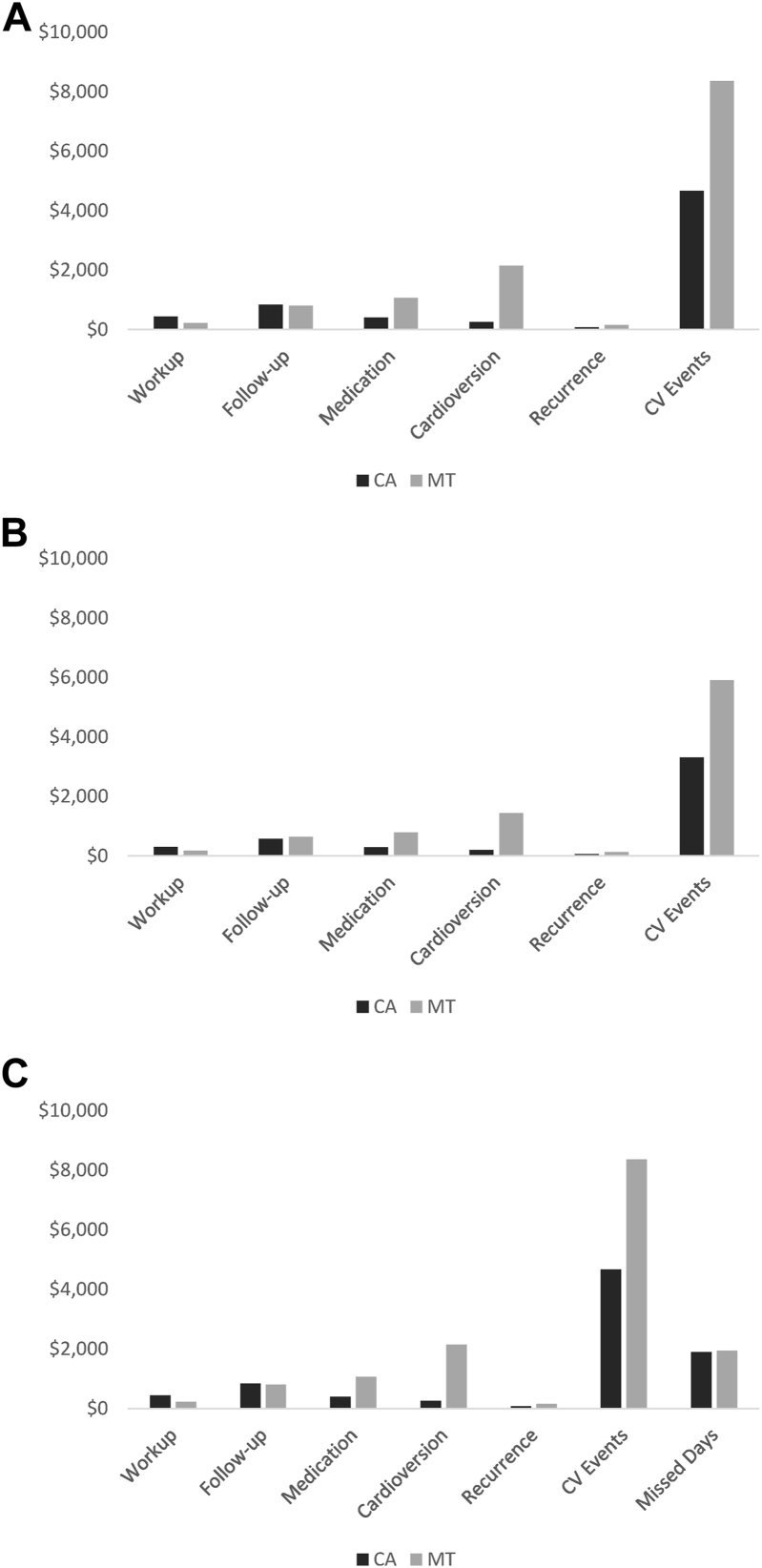
Summary of model costs. In addition to looking at the total costs, the breakdown of simulated costs by each category of utilization is shown. Outside of the procedural costs, cardiovascular (CV) hospitalizations were responsible for most of the costs incurred, accounting for 52% of the annual cost offset value for **A:** commercial payers and **B:** Medicare, and **C:** 44% for self-insured employers. In addition, cardioversions also represented a considerable portion of the annual costs. CA = catheter ablation; MT = medical therapy.

The breakdown of simulated costs by category of utilization is shown in Figure 3, as well as in the Supplemental Material (Supplemental Table 1). Other than procedural costs, CV hospitalizations were responsible for most of the costs incurred, accounting for 52% of the annual cost offset value for commercial payers and Medicare and 44% for self-insured employers. In addition, cardioversions also represented a considerable portion of the annual costs.

### Sensitivity analyses

When comparing RFCA and MT, 1-way sensitivity analysis revealed that the commercial and Medicare payer perspective models were most sensitive to the other or general CV hospitalizations rate for the MT group, ablation procedure cost, and other or general CV hospitalization rate for the RFCA group. The self-insured employer perspective model was most sensitive to the ablation procedure cost, the other or general CV hospitalization rate for the RFCA group, and the cardioversion rate. The tornado diagrams displaying the full results are shown in the Supplemental Material (Supplemental Figure 1).

The probabilistic sensitivity analysis showed a 95% confidence interval (CI) of 3.9 to 7.9 years around the break-even estimate for RFCA from a commercial payer perspective. Similarly, the 95% CI from the Medicare payer perspective to break even was determined to be 2.8 to 5.6 years. Finally, the 95% CI for time to the break-even point for RFCA costs was determined to be 3.6 to 6.7 years from the self-insured payer perspective. The full results of the sensitivity analysis can be found in the Supplemental Material (Supplemental Table 2).

### Scenario analyses: Inclusion of repeat ablation and MT-to-ablation crossover

The annual repeat ablation rate was 7.8%, per the PRECEPT clinical trial, while conversely it was estimated that, annually, 7.7% of patients in the MT arm would receive an ablation. Ultimately, there was a very marginal change from the base case results, in terms of difference in costs as well as the estimated break-even point, when including both repeat ablation and MT-to-ablation crossover.

The full results of this analysis are shown in Table 4. From the perspective of a commercial payer, the difference in total expected annual costs was $34,949 for RFCA vs MT, vs $34,916 in the base analysis, with an estimated break-even point of 5.9 years, which is exactly the same as the base case analysis.

**Table 4:** Scenario analysis: Repeat ablation and MT-to-ablation crossover

Strategy	Total cost	Incremental value	Offset/utilization only	Incremental value	Years until break-even
Commercial					
CA	$50,848	$34,949	$8594	–$7086	5.9
MT	$15,899	$34,916∗	$15,679	–$7118∗	5.9∗
Medicare					
CA	$27,036	$16,375	$5467	–$5021	4.3
MT	$10,661	$16,358∗	$10,488	–$5037∗	4.2∗
Self-insured employer					
CA	$52,751	$34,821	$9159	–$8369	5.2
MT	$17,930	$34,867∗	$17,528	–$8402∗	5.1∗

CA = catheter ablation; MT = medical therapy.

∗ Base case.

## Discussion

PsAF presents a significant burden to both patients and the health care system. Significant advances have been made over the years in RFCA technology, with studies such as the PRECEPT trial showing the potential clinical benefits of RFCA in PsAF populations. However, with growing constraints and scrutiny on health care spending, it is increasingly important to also consider the economic impact of treatments in new populations or with new technologies. This study sought to evaluate the annual costs and determine the cost offset and projected break-even point for patients receiving RFCA vs MT for PsAF.

### Key findings

Despite a higher initial cost, RFCA led to reduced health resource utilization over time, resulting in an annual cost offset valued at between $5037 and $8402, depending on the type of payer. Projecting the annual cost offset forward, RFCA costs are expected to break even after 4.2, 5.1, and 5.9 years for Medicare, self-insured employers, and commercial payers, respectively. The largest driver of the time to break even is the reduction in CV hospitalizations after RFCA, accounting for roughly 52% of the annual cost offset value for commercial payers and Medicare and 44% for self-insured employers. The sensitivity analyses showed that the model results are robust to expected changes in parameter values and are most sensitive to the ablation procedure cost and hospitalization rates. The scenario analysis showed that factoring in repeat ablation and crossovers only led to a very marginal change in the results. This is due to the fact that the repeat ablation rate and the MT-to-ablation crossover rate were nearly identical.

### Comparison with existing literature

There are few published economic models evaluating CA for patients with PsAF, as most are focused on paroxysmal AF. One study by Anderson and colleagues^30^ in 2014 found CA to be cost-effective compared with MT over a 5-year period in patients with nonparoxysmal AF. Moreover, in patients that were considered high risk for AF complications, CA led to lower costs at 5 years.

More generally, a study by Ladapo and colleagues^31^ in 2012 compared utilization and costs in the pre- vs postablation periods using a large retrospective claims database, using a similar structure to that in which the PRECEPT trial data were used in the current study, and found a decrease in costs after ablation. Anderson and colleagues^30^ found expenditures to be decreased significantly after ablation, with annual savings between $3300 and $9200. Additionally, savings were greater in the cohort 65 years of age or older and extended out to 3 years. A study by Mansour and colleagues^32^ in 2019 showed that ablation using contact force sensing technology led to cost savings over a 1-year period compared with ablations with non–contact force catheters. A study by Kleinman and colleagues^33^ found that CAs for AF led to decreased costs over a 36-month period for self-insured employers.

A recent cost-effectiveness study was conducted evaluating the data produced from the CABANA clinical trial.^34^ This study looked at patients over a 5-year time period and found higher costs in the ablation group. An important aspect to consider is that this study used intent-to-treat cohorts despite very substantial crossover in the CABANA trial. Many of the drug patients ultimately received ablations, which can impact their outcomes from that point forward. The authors noted that this was likely a large reason the differences between groups weakened after 90 days.

### Limitations

We acknowledge the economic model presented here is a simplistic representation of the real-world clinical practice and does not incorporate some of the complexities of patient care. The PRECEPT trial underlying the estimations in the model includes highly experienced and high-volume centers in which initial success may be higher and thus may differ from centers or operators with less experience.

The PRECEPT trial was a single-arm study; therefore, the MT arm was assumed to be the equivalent to the RFCA arm at baseline, with postbaseline costs derived from a supplemental literature search. As such, some of the comparisons in this analysis were based on indirect comparisons or were compiled from multiple studies. Repeat ablations and AF progression were not modeled; rather, the difference in treatment effects and associated cost differentials were assumed to remain constant over time. While we are aware that projecting a constant benefit over time is an oversimplification of the complexities seen in the real world, we do not believe that it caused a systemic bias in favor of ablation. It is important to consider that while the rates of recurrence and health care utilization may change, or increase, over time, if the difference between groups remains relatively constant, the results of this model remain valid.

The base case model did not include repeat ablations or patients in the MT arm crossing over to receive an ablation. This was a simplifying assumption to show the specific impact of using CA, as opposed to just MT. However, the scenario analysis evaluated the inclusion of these events and showed that there would be minimal impact on the results, due to the fact that the rates of these events were very similar. Furthermore, we believe that the real-world rates of MT-to-ablation crossover would likely exceed the repeat ablation rate, and consequently, we believe that these analysis results are conservative and the subsequent conclusions valid.

Finally, this study explicitly evaluated the financial impact in terms of cost; however, another important consideration is the value and cost-effectiveness of an intervention. While the data to evaluate cost-effectiveness were not available in the PRECEPT trial, this is an important topic for future studies to explore.

## Conclusion

The current study shows that, in addition to providing clinical benefits, RFCA may be a valuable economic investment for U.S. payers. Despite the higher initial cost, it leads to substantially reduced health care utilization, with a projected break-even time of approximately 4 to 6 years for U.S. payers.

## Funding Sources

This work was supported by 10.13039/100007497Biosense Webster, Inc.

## Disclosures

Drs Imhoff and Hunter are employees of CTI Clinical Trial & Consulting Services, who are consultants to 10.13039/100007497Biosense Webster, Inc, the study sponsor. Drs Maccioni and Wei are paid employees of Johnson and Johnson. Dr Osorio is a paid consultant and has received research grants from 10.13039/100007497Biosense Webster. Dr Mansour has served as a consultant for Biosense Webster, Abbott, Medtronic, Boston Scientific, Janssen, Philips, Novartis, andSentreHEART; has received research grants from Biosense Webster, Abbott, Boston Scientific, Medtronic, Pfizer, Boehringer Ingelheim; and has an equity interest in EPD Solutions, NewPace Ltd, and Affera. Dr Melby is a paid consultant and has received research grants from 10.13039/100007497Biosense Webster. Dr Natale is a paid consultant for Abbott, Baylis, Boston Scientific, Biosense Webster, Biotronik, Medtronic.

## Authorship

All authors attest they meet the current ICMJE criteria for authorship.

## Ethics Statement

This analysis is based primarily on previously published data, though supplementary analyses were performed using additional de-identified data from patients in the PRECEPT trial that already consented to the use of their trial data; thus, this study is exempt from institutional review board review.
