# Supplementary material for: Economic Evaluation of Contact Force Catheter Ablation for Persistent Atrial Fibrillation in the United States
Source: Heart Rhythm O2. 2022 Sep 20;3(6Part A):647–55. doi: 10.1016/j.hroo.2022.09.011 (PMC9795304; doi:10.1016/j.hroo.2022.09.011)
Supplement: Supplemental Material [file mmc1.docx]

**Table S1. Model Results by Utilization Category**

| **Parameter** | **Commercial** | | **Medicare** | | **Self-Insured Employers** | |
| --- | --- | --- | --- | --- | --- | --- |
|  | **CA^*^** | **MT^†^** | **CA** | **MT** | **CA** | **MT** |
| Workup | $441 | $220 | $295 | $173 | $441 | $220 |
| Procedure | $40,975 | NA | $20,699 | NA | $40,975 | $0 |
| Follow-up | $839 | $801 | $574 | $635 | $839 | $801 |
| Medication | $396 | $1,061 | $289 | $792 | $396 | $1,061 |
| Cardioversion | $258 | $2,146 | $202 | $1,440 | $258 | $2,146 |
| Recurrence | $73 | $152 | $58 | $120 | $73 | $152 |
| CV^‡^ Events | $4,670 | $8,356 | $3,304 | $5,902 | $4,670 | $8,356 |
| Stroke | $392 | $719 | $277 | $509 | $392 | $719 |
| Bleeding events | $738 | $720 | $522 | $510 | $738 | $720 |
| Cardiac arrest | $0 | $117 | $0 | $70 | $0 | $117 |
| Other | $3,540 | $6,800 | $2,504 | $4,814 | $3,540 | $6,800 |
| Missed days from Work | N/A | N/A | N/A | N/A | $1,902 | $1,951 |

^*^ Catheter ablation

^†^ Medical therapy

^‡^ Cardiovascular

**Table S2. Probabilistic Sensitivity Analysis**

| **Strategy** | **Total Cost** | **Incremental Value** | **Offset / Utilization Only** | **Incremental Value** | **Years Until Break-even** | **95% Confidence Range** |
| --- | --- | --- | --- | --- | --- | --- |
| Commercial |  |  |  |  |  |  |
| Catheter ablation | $47,648 | $34,911 | $5,396 | -$7,121 | 5.9 | 3.9 to 7.9 years |
| Medical therapy | $12,737 |  | $12,517 |  |  |  |
| Medicare |  |  |  |  |  |  |
| Catheter ablation | $25,412 | $16,351 | $3,852 | -$5,037 | 4.2 | 2.8 to 5.6 years |
| Medical therapy | $9,061 |  | $8,889 |  |  |  |
| Self-insured employer |  |  |  |  |  |  |
| Catheter ablation | $49,556 | $34,870 | $5,962 | -$8,401 | 5.2 | 3.6 to 6.7 years |
| Medical therapy | $14,686 |  | $14,363 |  |  |  |

**Figure S1. One-Way Sensitivity Analysis Results**

Each parameter in the model was varied individually to measure the effect on the results. The variation from the base model results are shown in the tornado diagrams for each payer. When comparing RFCA and MT, the commercial (**S1a**) and Medicare (**S1b**) payer perspective models were most sensitive to the other/general CV hospitalizations rate for the MT group, ablation procedure cost, and other/general CV hospitalization rate for the RFCA group. The self-insured employer (**S1c**) perspective model was most sensitive to the ablation procedure cost, the other/general CV hospitalization rate for the RFCA group, and the cardioversion rate.

**Figure S1a. One-Way Sensitivity Analysis Results — Commercial Payer**

**Figure S1b. One-Way Sensitivity Analysis Results — Medicare**

**Figure S1c. One-Way Sensitivity Analysis Results — Self-Insured Employer**
